# Assessing pharmacy staff practices and knowledge of off-label drug use in Ministry of Health Hospitals, Saudi Arabia: A cross-sectional study

**DOI:** 10.1097/MD.0000000000044015

**Published:** 2025-08-29

**Authors:** Mohammed A. Alomary, Norah F. Alshehri, Abdullah M. Alzahrani, Ayed A. Alkatheeri, Maan H. Harbi, Migahed H. Almalki, Najwa A. Almutairi, Shikhah G. Aldajani, Dunya M. Emam, Rayan M. Alzahrani, Mohammed O. Almalki, Yahya A. Alzahrani

**Affiliations:** a Department of Pharmacy, King Fahad General Hospital, Ministry of Health, Jeddah, Saudi Arabia; b Department of Pharmacy, Clinical pharmacy unit, East Jeddah Hospital, Ministry of Health, Jeddah, Saudi Arabia; c Pharmaceutical Care Department, Ministry of National Guard—Health Affairs, Jeddah, Saudi Arabia; d King Abdullah International Medical Research Center, Jeddah, Saudi Arabia; e Department of Pharmacy, Drug Information Center, East Jeddah Hospital, Ministry of Health, Jeddah, Saudi Arabia; f Pharmacology and Toxicology Department, College of Pharmacy, Umm Al-Qura University, Makkah, Saudi Arabia; g Department of Pharmacy, East Jeddah Hospital, Ministry of Health, Jeddah, Saudi Arabia; h Sales Department, Boehringer Ingelheim, Jeddah, Saudi Arabia; i Department of Pharmacy, Riyadh Long-Term Care Hospital, Ministry of Health, Riyadh, Saudi Arabia; j Department of Pharmacy, AL Nahdi Medical Company, Jeddah, Saudi Arabia; k College of Pharmacy, Albaha University, Albaha, Saudi Arabia; l Department of Pharmacology, Faculty of Medicine, King Abdul-Aziz University, Rabigh, Saudi Arabia.

**Keywords:** healthcare reform, medication safety, off-label drug use, pharmacist knowledge, pharmacist practice, regulatory awareness, Saudi Arabia

## Abstract

Despite the growing prevalence of off-label prescribing, there is limited research exploring pharmacists’ actual knowledge and practices, particularly in the Saudi context. Existing literature mainly focuses on physicians, leaving a gap in understanding pharmacists’ readiness to handle such prescriptions. This study aimed to assess the knowledge and practices of pharmacy staff in Saudi Arabia regarding off-label drug use and examined the impact of education, age, gender, and experience on these outcomes. A cross-sectional survey was conducted over 6 months (February–July 2024) targeting registered pharmacy staff working in healthcare settings in Riyadh and Jeddah, Saudi Arabia. Participants completed a content validated, structured questionnaire designed to assess their knowledge and practices related to off-label drug use. The survey also captured demographic and professional data, including age, gender, education level, hospital type, and years of experience. Among 302 pharmacy staff, the mean knowledge score was 54.7 ± 22.3, indicating moderate knowledge while Practice scores were higher (mean 68.2 ± 17.8), with over 75% correctly managing off-label prescriptions. Logistic regression showed hospital level as a significant predictor, staff in tertiary hospitals were more knowledgeable (OR = 4.5) and practiced better (OR = 3.6). Demographics had no significant impact. No association was found between self-rated knowledge and actual knowledge, but poor self-rated familiarity strongly correlated with poor practice (*P* < .0001). These findings underscore the need for SFDA-aligned training and regulatory initiatives to improve off-label prescribing, especially in primary and secondary care. Periodic national assessments are essential to monitor and enhance pharmacy staff competence amid ongoing healthcare reforms.

## 1. Introduction

Off-label use of medications has been a long-standing practice, primarily driven by therapeutic advantages, liability implications, and a strong focus on patient safety. In the 1960s, the Thalidomide disaster, which caused severe birth defects and numerous deaths, marked a turning point in the regulation of the pharmaceutical and medical sectors.^[1]^ Addressing unmet medical needs through conventional treatment techniques is possibly the most significant aspect of off-label practice, offering key advantages such as increased access to medications for specific patient populations. According to the existing literature, off-label use is particularly prevalent in the management of rare diseases. This approach facilitates the exploration of alternative treatment options when standard therapies are insufficient or unavailable. Despite the potential benefits, it is crucial to balance off-label prescribing with rigorous evaluation, clinical trials, and ongoing monitoring to ensure the safety and efficacy of medications.^[2]^ The lessons learned from the tragedy of Thalidomide emphasize the importance of evidence-based medicine and the need for robust regulatory frameworks to protect patient well-being. Off-label drug use refers to “drugs prescribed and used outside their licensed indications with respect to dosage, age, indication, or route.”^[3]^ In contrast, unauthorized or unlicensed use occurs when a drug is used in a country without obtaining marketing authorization from the respective licensing authority, even if the drug is licensed elsewhere.^[4]^ Marketing authorization holders are granted permission to commercialize the drug product within specific restrictions set by regulatory bodies, such as national drug agencies, the European Medicines Agency (EMA), and the U.S. Food and Drug Administration (FDA).^[5]^

In many countries, specific guidance on off-label medical usage is scarce, and regulatory consistency across nations is limited. Regulatory authorities typically neither endorse nor prohibit off-label use. The FDA mandates that physicians and pharmacists employ legally approved drugs in accordance with good medical and pharmaceutical practice.^[6]^ When prescribing outside the Summary of Product Characteristics (SmPC), the prescriber must possess thorough knowledge of the product. Clinicians should conduct comprehensive assessments, considering potential risks and adverse effects before prescribing off-label.^[7]^ The FDA supports off-label use following these requirements, and the American Medical Association (AMA) views it as an innovation and the physician’s prerogative for patient benefit.^[8]^ Off-label prescribing and dispensing do not constitute clinical research. In Europe, safety enhancement and risk reduction are prioritized, including in off-label scenarios. European pharmaceutical legislation neither prohibits nor permits off-label practice, and physicians can prescribe off-label drugs at their discretion.^[9]^ This stance facilitates access to medications proven safe and effective. In India, pharmaceutical companies cannot advertise unapproved indications, but physicians may prescribe approved drugs for unapproved indications based on emergencies, lack of approved treatments, or perceived benefits from previous studies.^[10]^

Off-label drug use can span multiple categories, such as indication and dosage, for a single drug component. Rare diseases present challenges for clinical trials and drug development due to small patient populations and high costs.^[2]^ Consequently, most treatments for rare diseases are provided off-label. In cancer research, off-label use is prevalent for both rare and common cancers, with some studies estimating that 50% of cancer therapy is administered off-label. For pediatric patients, off-label prescriptions are common due to a lack of pediatric formulations.^[11,12]^ Several studies reported off-label prescription rates ranging from 1.2% to 99.7%.^[7]^ In psychiatry, off-label use is well-documented, targeting specific patient populations such as pediatric and geriatric patients. Monitoring the safety of off-label use is crucial, as it may be the only treatment option for certain patients.^[6]^ Various methods for monitoring off-label use include unexpected reports, observational pharmacoepidemiologic studies, databases, and clinical trials. Adverse effects of medications are often documented in pharmacovigilance databases, including those related to off-label use. However, concerns of underreporting exist within national spontaneous reporting systems. In conclusion, off-label drug use is prevalent across multiple therapeutic areas and patient populations, emphasizing the importance of proper monitoring and reporting to ensure patient safety.^[13]^

In the Kingdom of Saudi Arabia, the Saudi Food and Drug Administration (SFDA) mandates permission for all medications to ensure efficacy, safety, and quality. Despite this, there is no definitive description for off-label drug use provided by the Saudi Ministry of Health or the SFDA.^[14]^ Healthcare settings in the kingdom must adhere to the Saudi Central Board for Accreditation of Healthcare Institutions (CBAHI) standards, which include a system for prescribing non-formulary medications and formulary medications for off-label indications or investigations.^[15]^ A 12-month retrospective analysis conducted in 6 tertiary hospitals in Saudi Arabia revealed a high prevalence of off-label prescriptions, particularly in adults and geriatrics with depression and diabetes mellitus. Some authors suggest publishing off-label therapy guidelines to improve safety and ensure the relevance of referenced studies. Pharmacists must be aware of concerns surrounding off-label prescribing in order to make informed decisions regarding such practices.^[16]^

In light of the increasing responsibility of pharmacists in safeguarding public health and ensuring safe drug usage, understanding the concerns related to off-label prescribing is essential for making informed decisions in clinical practice. This study aims to build upon existing research by examining the knowledge, attitudes, and practices of Saudi Arabian pharmacists regarding off-label drug prescription. The importance of this investigation stems from the fact that most of the previous research on off-label medication prescription has been conducted in the USA, the United Kingdom, and other European countries, leaving a gap in understanding the context within Saudi Arabia. The objective of this study is to evaluate the knowledge, attitudes, and current practices of Saudi hospital pharmacists focusing on dispensing off-label medications, thereby contributing to a more comprehensive understanding of this issue in different healthcare settings.

## 2. Materials and methods

### 2.1. Study place, design, setting, and patient population

This study was conducted over a 6-month period (February–July 2024). The questionnaire was initiated by the study team after comprehensive evaluation of the literature [21–22]. The instrument comprises 3 sections: participant demographics, assessment of pharmacy staff knowledge, and evaluation of pharmacy care providers’ practices regarding off-label drug use. The questionnaire consists of 21 items, with the cumulative score representing the number of correct responses. Data collection was facilitated through an anonymous online survey platform (Google Forms). Potential participants were presented with an informed consent statement in the survey header, which they must acknowledge to confirm their eligibility and voluntary participation in the study. The questionnaire’s content validity was established through expert review (Survey final, Supplemental Digital Content, https://links.lww.com/MD/P837).

The questionnaire was developed to assess distinct and independent aspects of off-label drug use, including regulatory understanding, clinical judgment, and institutional practices. As it was designed to capture a broad range of content rather than measure a single latent construct, internal consistency was not a primary design criterion. This approach reflects the nature of knowledge- and practice-based assessments, where variability across item content is both expected and necessary to ensure comprehensive domain coverage.

### 2.2. Inclusion and exclusion criteria

The study population consists of registered pharmacy care providers who are actively employed in the healthcare setting within Saudi Arabia. Eligibility for participation requires that individuals must be actively working in their professional capacity at the time of the study. Exclusions from the study include pharmacy care providers who are not registered with the appropriate regulatory bodies, individuals working outside the specified geographical locations of Riyadh and Jeddah, and participants who were involved in the pilot phase of the study.

### 2.3. Sample size calculation

The sample size was calculated using the single population proportion formula:

$n=\frac{z{\left(\propto /2\right)}^{2\;}\times P\left(1-P\right)}{D^{2}}$ where *n* is the required sample size, *Z(α/2*) is the standard normal value at a 95% confidence level (1.96), *P* is the assumed population proportion (0.5, to maximize sample size), and *D* is the margin of error (0.05).

Substituting these values, the estimated sample size was:


$$\begin{array}{l} n=\frac{1.96^{2\;}\times 0.5\times \left(1-0.5\right)}{0.05^{2}}=384 \end{array}$$

### 2.4. Ethical consideration

The study was approved by the Institutional Review Board of the Directorate of Health Affairs in Jeddah, Saudi Arabia, which is nationally registered with the National Committee of Bioethics at King Abdulaziz City for Science and Technology (NCBE-KACST), under registration number H-02-J-002.

### 2.5. Endpoints

#### 2.5.1. Primary endpoint

To assess the knowledge and current practices of Saudi hospital pharmacists regarding the dispensing of off-label medications.

#### 2.5.2. Secondary endpoint

To evaluate the impact of demographic and professional characteristics – specifically education level, age, gender, and practical experience – on pharmacists’ knowledge and practices related to off-label drug use.

### 2.6. Scoring

To categorize participants’ knowledge of off-label drug use, we employed a median split approach. The total knowledge scores were calculated for each participant based on their correct responses to the assessment items. The median knowledge score across all participants was determined to be 35. Participants who scored 35 or higher were classified as “Knowledgeable,” indicating a higher level of understanding of off-label drug use. Conversely, participants with scores below 35 were classified as “Not Knowledgeable,” reflecting a relatively lower level of understanding. This classification allowed us to dichotomize the participants into 2 distinct groups for further analysis, facilitating the exploration of factors associated with varying levels of knowledge regarding off-label drug use. On the other hand, to evaluate the participants’ practices concerning off-label drug use, we calculated a practice score for each participant based on their responses to a series of relevant questions. The median practice score across the study cohort was identified as 15. Participants who achieved a score of 15 or higher were classified as exhibiting “Good Practice,” indicating adherence to recommended practices regarding off-label drug use. Those scoring below 15 were classified as exhibiting “Poor Practice,” suggesting a need for improvement in their approach to off-label drug use. This classification enabled the segmentation of participants into 2 distinct groups for further analysis, allowing for a deeper investigation into the factors that contribute to effective or suboptimal practices in the context of off-label drug use.

### 2.7. Statistical analysis

All statistical analyses were performed using IBM SPSS Statistics version 26 (SPSS Inc., Chicago). Descriptive statistics were used to summarize participant characteristics, with continuous variables expressed as means and standard deviations and categorical variables as frequencies and percentages. Differences in mean knowledge and practice scores between groups were analyzed using independent *t*-tests and one-way ANOVA, as appropriate. Logistic regression analysis was conducted to identify demographic and institutional predictors of knowledge and practice levels, based on dichotomized scores. Additionally, chi-square tests were used to assess associations between self-perceived understanding and actual knowledge and practice classifications. A *P*-value of <.05 was considered statistically significant.

## 3. Results

### 3.1. Content validity index (CVI) analysis

The Content validity ratio (CVR) was calculated for each item based on the ratings provided by 9 experts, according to Lawshe method. The CVR values indicate the extent to which each item is deemed essential by the panel of experts. The CVR values for the items ranged from 0.333 to 1.000, reflecting varying degrees of agreement among the experts regarding the necessity of each item. The overall content validity index (CVI), representing the average CVR across all items, was calculated to be approximately 0.752. This CVI value suggests a high level of agreement among the experts that the items included in the questionnaire are essential and necessary for the intended assessment.

### 3.2. Participant characteristics

The demographic characteristics of the study population are summarized in Table 1. A total of 302 participants were included, with a male predominance (58.9%). The majority of the participants were aged between 30 to 50 years (81.1%), with only a small fraction aged over 50 years (1.3%). In terms of educational level, the most common qualification was pharmacy (64.2%), followed by pharmacy technician (20.9%). The participants were primarily employed in tertiary hospitals (71.2%), with the Western region being the most represented geographic area (65.2%). Regarding professional experience, a substantial proportion of the respondents had 10 to 14 years of experience (37.1%), while only a small group had over 20 years of experience (1.7%).

**Table 1:** Demographic and professional characteristics of the study participants.

Character	Frequency (n)	Percentage (%)
Gender		
Male	178	58.9
Female	124	41.1
Age (yr)		
≤30	53	17.5
>30 to ≤50	245	81.1
>50	4	1.3
Educational level		
Pharmacist	194	64.2
Senior pharmacist	41	13.6
Consultant pharmacist	4	1.3
Pharmacist technician	63	20.9
Level of hospital		
Primary	36	11.9
Secondary	51	16.9
Tertiary	215	71.2
Region		
Western	197	65.2
Central	68	22.5
Eastern	1	0.3
Northern	2	0.7
Southern	34	11.3
Years of experience		
0–4 yr	41	13.6
5–9 yr	104	34.4
10–14 yr	112	37.1
15–19 yr	40	13.2
≥20 yr	5	1.7

### 3.3. Pharmacy staff knowledge of off-label drug use

The mean knowledge score was 54.7 ± 22.3 out of 100, indicating moderate knowledge levels regarding off-label drug use. As shown in Table 2, the majority demonstrated strong understanding of fundamental concepts: 87.7% (n = 265) correctly identifying the definition of off-label drug use, and 75.5% (n = 228) recognizing its commonality in practice. Notably, 79.5% (n = 240) of respondents accurately acknowledged that off-label drug use could become a widely accepted practice or standard of care. However, knowledge gaps were evident in certain areas. Only 26.5% (n = 80) correctly identified the age group where off-label drug use is most common, while almost half of participants (54.6%, n = 165) could accurately pinpoint references indicating off-label use. Regarding safety and regulatory aspects, 71.9% (n = 217) correctly identified the major concern of increasing off-label drug use, while 61.6% (n = 186) demonstrated understanding of the safety implications of prescribing off-label drugs. Interestingly, 80.5% (n = 243) recognized the inadequacy of available risk/benefit information for off-label drug use. Knowledge of regulatory and promotional aspects was mixed. While 71.9% (n = 217) were aware of the role of Pharmacy and Therapeutic Committees, only 45% (n = 136) correctly understood regulations regarding pharmaceutical company promotions for unapproved indications.

**Table 2:** Pharmacy staff knowledge of off-label drug use.

Item No.	Knowledge statement	True, n (%)
1	Off-label drug use is	265 (87.7)
2	Off-label drug use is common	228 (75.5)
3	Can an off-label drug use for a given drug become a widely accepted practice or standard of care?	240 (79.5)
4	In which age group did you think off-label drug use is most common?	80 (26.5)
5	Which of the following reference indicates that the use of a drug is considered off-label?	165 (54.6)
6	The major concern regarding the trending increase of off-label drug use is:	217 (71.9)
7	In your opinion, how safe is prescribing off-label drugs?	186 (61.6)
8	The risk/benefit information available on the off-label use of drugs is appropriate	59 (19.5)
9	Pharmacy and Therapeutic (P&T) Committee	217 (71.9)
10	Can a pharmaceutical company make a promotion for an unapproved indication?	136 (45.0)
11	Can a pharmaceutical company speak about off-label use during CME activities?	82 (27.2)
12	Insurance companies easily pay for off-label use	114 (37.7)
13	If a drug is effective, the major obstacle to obtaining government approval to convert its off-label uses to on-label uses is	159 (52.6)
	Mean score ± SD	54.7 ± 22.3

### 3.4. Pharmacy staff practices related to off-label drug use

The mean practice score was 68.2 ± 17.8 out of 100, suggesting a higher level of practical understanding compared to theoretical knowledge. As shown in Table 3, a substantial majority (75.5%, n = 228) recognized pharmacists’ authority to refuse inappropriate prescriptions, even against physician insistence. Similarly, 75.8% (n = 229) indicated they would take appropriate action upon receiving an off-label prescription. Practical knowledge of specific off-label uses was high, with 79.8% (n = 241) correctly identifying gabapentin’s off-label use in diabetic neuropathy. However, only 41.7% (n = 126) were aware of existing laws or regulations regarding off-label drugs in Saudi Arabia issued by the Saudi Food and Drug Authority (SFDA).

**Table 3:** Pharmacy staff practices related to off-label drug use.

Item No.	Practice statement	True, n (%)
1	Pharmacists have the authority to refuse to fill a prescription if they believe it is incorrect, even if a physician insists on it.	228 (75.5)
2	If you received an off-label prescription, the appropriate action will be taken is	229 (75.8)
3	Gabapentin has been labeled used in postherpetic neuralgia while it is used as an off-label in treatment of the diabetic neuropathy	241 (79.8)
4	There is a law or regulation regarding off-label drugs in the Kingdom of Saudi Arabia released by the SFDA	126 (41.7)
	Mean score ± SD	68.2 ± 17.8

### 3.5. Impact of hospital level and demographic factors on pharmacy staff knowledge of off-label drug use

A logistic regression analysis was performed to evaluate the influence of demographic and professional factors on the likelihood of healthcare professionals being knowledgeable about off-label drug use. The model incorporated gender, age, educational level, the level of the hospital where the participant works (coded as 1 for Primary, 2 for Secondary, and 3 for Tertiary), and years of experience as predictors. The analysis revealed that the level of the hospital was a significant predictor of knowledge regarding off-label drug use. Specifically, healthcare professionals working at tertiary hospitals were 4.5 times more likely to be knowledgeable compared to those working at primary hospitals (Exp(B) = 4.504, *P* < .001). Additionally, professionals at secondary hospitals were 2.4 times more likely to be knowledgeable compared to those at primary hospitals (Exp(B) = 2.427, *P* = .007). Other variables, including gender, age, educational level, and years of experience, were not statistically significant in predicting knowledge about off-label drug use. For example, gender (Exp(B) = 1.474, *P* = .170), age categories (Exp(B) = 0.184, *P* = .240 for Age 1; Exp(B) = 0.251, *P* = .299 for Age 2), and educational level (Exp(B) = 0.644, *P* = .185 for educational level 1) did not show significant associations with the likelihood of being knowledgeable.

### 3.6. Determinants of off-label drug use practices among pharmacy staff: role of hospital level and demographics

A logistic regression analysis was conducted to investigate the factors influencing healthcare professionals’ practice related to off-label drug use. The independent variables included gender, age, educational level, level of hospital (coded as 1 for Primary, 2 for Secondary, and 3 for Tertiary), and years of experience.

The analysis identified the level of hospital as a significant predictor of practice regarding off-label drug use. Specifically, professionals working at tertiary hospitals were 3.6 times more likely to engage in appropriate off-label drug use practices compared to those at primary hospitals (Exp(B) = 3.649, *P* = .001). Similarly, those working at secondary hospitals were 3.5 times more likely to exhibit appropriate practices compared to those at primary hospitals (Exp(B) = 3.517, *P* < .001).

Other variables, including gender (Exp(B) = 1.346, *P* = .282), age (Exp(B) = 0.000, *P* = .999 for both age groups), educational level (Exp(B) = 1.237, *P* = .544 for Educational level 1; Exp(B) = 2.299, *P* = .069 for Educational level 2), and years of experience, did not show statistically significant associations with the likelihood of appropriate off-label drug use practices.

### 3.7. Discrepancy between perceived and actual competence in off-label drug use among pharmacy staff

**Figure 1.**
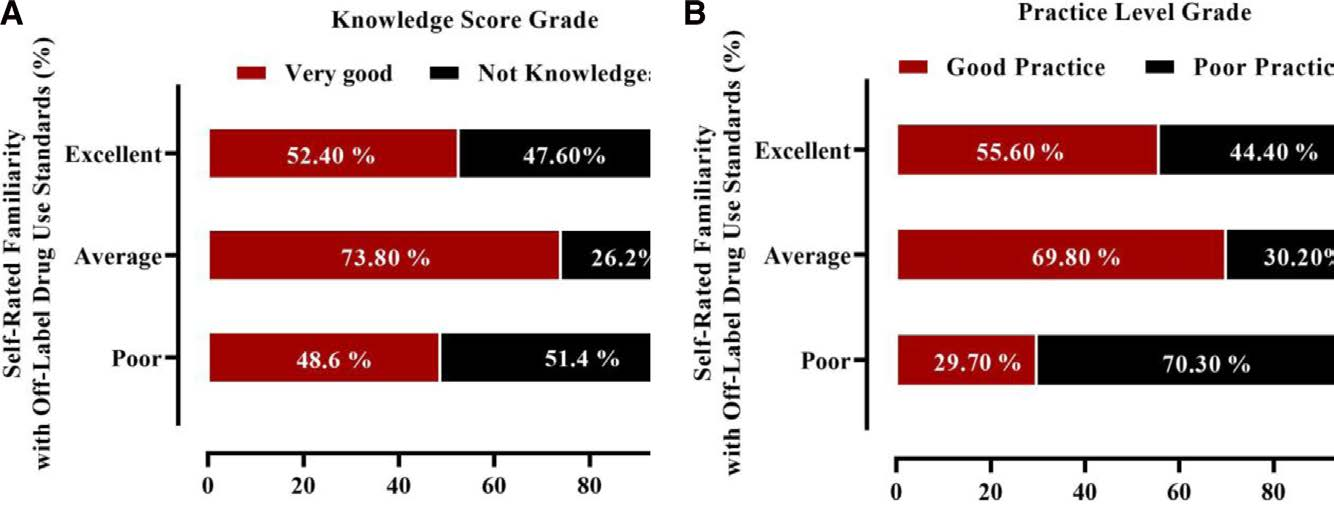
Self-rated familiarity with off-label drug use standards in relation to (A) knowledge score results and (B) practice score results. Statistical comparison was performed using the chi-square test; *P* < .05 was considered statistically significant.

The analysis of the relationship between self-rated understanding of off-label drug use and knowledge score results revealed no significant association between these variables. The chi-Square test yielded a *P*-value of 0.106, indicating that there is no statistically significant relationship between participants’ self-assessed understanding of off-label drug use and their knowledge score results “Knowledgeable” or “Not Knowledgeable.” post hoc analysis using standardized residuals further corroborated this finding, with no significant deviations detected in the cross-tabulation after applying the Bonferroni correction. In contrast, the analysis of the relationship between self-evaluated familiarity with standards for off-label drug use and practice score results revealed a significant association. Participants who rated their familiarity as “Poor” were significantly more likely to be classified as engaging in “Poor Practice” concerning off-label drug use, as indicated by a highly significant residual (*P* < .0001). This finding implies that individuals who perceive themselves as having an inadequate understanding of the standards are more prone to exhibit suboptimal practices in managing off-label prescriptions. Conversely, no significant associations were observed between self-evaluations of “Excellent” or “Average” familiarity and practice score results, as shown in Figure 1.

## 4. Discussion

Our survey revealed that pharmacy staff in Saudi Arabia demonstrated good knowledge, attitudes, and practices regarding off-label drug use in healthcare settings. Thus, this study reinforces previous findings and adds new evidence regarding pharmacy staff knowledge, attitudes, and practices toward off-label drug use.

The mean knowledge score (54.7 ± 22.3) indicates a moderate level of understanding of off-label drug use concepts by the participants, aligning with findings from other studies. In 2 studies, at least half of healthcare professionals, including pharmacists, could define off-label drug use, though only 37% felt confident in their knowledge when it came to the pediatric population.^[17,18]^ This suggests that while many pharmacists may know the concept, they may need a more comprehensive understanding and training. However, our study uncovered specific knowledge deficits. Only 26.5% correctly identified pediatrics as the age group with the highest rates of off-label prescribing, as highlighted by Petkova et al (2023).^[7]^ This emphasizes the need for targeted education in pediatric pharmacotherapy.

A critical finding with significant implications for the changing health system in Saudi Arabia is the insurance coverage of off-label medications. Our study showed that 62.3% of respondents believed that insurance companies cover such drug usage. The knowledge gap is critical during the current Saudi government shift to privatized healthcare under Vision 2030, where reimbursement clarity is essential. This finding is fitting with Sheblaq et al (2019), who identified insurance ambiguity as a barrier to off-label prescribing in Saudi healthcare settings.^[16]^ Moving to an insurance-based healthcare model must drive more extensive knowledge of coverage limits and reimbursement guidelines among healthcare providers.^[19,20]^

One of the key findings of our analysis is the significant effect of institutional settings on knowledge and practice patterns. Pharmacy staff in tertiary hospitals were 4.5 times more likely to have higher knowledge scores than those in primary care, independent of education and experience. This supports previous findings that tertiary care pharmacists are more involved in benefit–risk assessments and reporting adverse events related to off-label use.^[21]^

The practice domain scores (68.2 ± 17.8) surpassed knowledge scores, suggesting that practical competence may develop more from experience than formal study. This was emphasized by Dresser and Frader (2009), who highlighted the value of clinical judgment in off-label decision-making.^[8]^ Encouragingly, 75.5% of participants agreed that pharmacists have a right and responsibility to reject inappropriate prescriptions, but should do so ethically, legally, and collaboratively.^[17,22,23]^

Our results strongly pinpoint specific regulatory knowledge deficits, since only 41.7% of respondents were familiar with SFDA regulations. Further, the strong institutional effect on both knowledge and practice indicates that, in practice, interventions would be more effective when carried out at the organizational level rather than at the individual practitioner level.

The fact that demographic variables such as gender, age, and number of years worked were not seen to have any influence on knowledge or practice scores contrasts with several other findings in related literature. For instance, Vilhelmsson et al found that professional experience was significantly associated with off-label prescription.^[9]^ This may be because, until 3 years ago, it was most practically done due to the standardized influence of institutional protocols and guidelines in Saudi healthcare settings, as indicated by more recent work from Alsaedi et al (2023).^[15]^

There was an interesting disparity between the self-assessed levels of understanding and knowledge (*P* = .106), with those acknowledging limited understanding also demonstrating substandard practices (*P* < .0001). This might indicate the presence of some degree of overconfidence in some practitioners. Wittich et al (2012) noted this in their observations of disparities in healthcare professionals’ self-assessments regarding competence in off-label prescribing.^[3]^ This indicates that some practitioners accurately recognize their knowledge limitations.

However, our findings are essential for policy and practice in developing off-label prescribing competency programs. The program should concentrate on establishing institutions, especially in primary care settings. As healthcare reform in Saudi Arabia continues, knowledge of health insurance coverage policies for off-label drugs becomes more critical to ensure smooth patient care and prevent conflicts between clinical decisions and health insurance coverage policies. Further research is required to understand how the tertiary hospital environment channels superior knowledge and practice patterns and how best to develop improved educational interventions across all healthcare settings.

This study is limited by its sampling method. Moreover, although Riyadh and Jeddah provide diverse and representative healthcare settings, the restriction to these cities may limit the generalizability of findings to other regions in Saudi Arabia. Future studies should aim to include additional regions to enhance external validity. The self-reported nature of the data also introduces potential response bias, especially since there seems to be a gap between self-perceived and actual knowledge levels. Additionally, the exclusive use of quantitative methods precludes a deeper understanding of the underlying causes of knowledge and practice gaps. Incorporating qualitative approaches, such as interviews or focus groups, could help uncover these underlying factors. Finally, being cross-sectional, it does not provide room for establishing causality or monitoring changes that take place over time regarding knowledge and practice, which may be highly relevant considering ongoing reforms to the health system in Saudi Arabia.

## 5. Conclusions

Pharmacy staff showed moderate knowledge but relatively stronger practices regarding off-label drug use, with notable gaps in regulatory understanding and pediatric prescribing. Tertiary hospital pharmacists demonstrated better knowledge and practice than those in primary care, while demographic factors had no significant influence. A clear mismatch was found between self-rated and actual scores, with poor self-perception of regulatory familiarity linked to suboptimal practices.

These findings emphasize the need for targeted institutional-level training and regulatory awareness – particularly in primary and secondary care settings – to enhance the safe and effective application of off-label prescribing. Implementing structured, SFDA-aligned educational programs could bridge regulatory knowledge gaps and promote safer prescribing behaviors. Future national policy should also mandate periodic assessments to monitor pharmacists’ competence in handling off-label use, especially in the evolving healthcare landscape of Saudi Arabia.

## Author contributions

**Conceptualization:** Mohammed A. Alomary.

**Data curation:** Mohammed A. Alomary, Rayan M. Alzahrani, Mohammed O. Almalki.

**Formal analysis:** Najwa A. Almutairi, Shikhah G. Aldajani, Yahya A. Alzahrani.

**Methodology:** Abdullah M. Alzahrani, Yahya A. Alzahrani.

**Resources:** Ayed A. Alkatheeri, Migahed H. Almalki.

**Supervision:** Yahya A. Alzahrani.

**Validation:** Norah F. Alshehri, Maan H. Harbi, Dunya M. Emam.

**Writing – original draft:** Norah F. Alshehri.

**Writing – review & editing:** Yahya A. Alzahrani.

## Supplementary Material


