# Supplementary material for: Assessing pharmacy staff practices and knowledge of off-label drug use in Ministry of Health Hospitals, Saudi Arabia: A cross-sectional study
Source: Medicine (Baltimore). 2025 Aug 29;104(35):e44015. doi: 10.1097/MD.0000000000044015 (PMC12401448; doi:10.1097/MD.0000000000044015)
Supplement: Supplementary file 1 [file medi-104-e44015-s001.docx]

**Section 1: Demographic**

**Please tick (X) in the appropriate answer box ⌧ or fill in the provided space when required.**

1. **Gender**

**⃣** Male ⃣ Female

1. **Age**

**⃣** < 30 years ⃣ 31 – 50 years ⃣ > 50 years

1. **Educational level?**

⃣ Consultant pharmacist

⃣ Senior Pharmacist

⃣ Pharmacist

⃣ Pharmacist technician

1. **Level of hospital**

**⃣** Primary ⃣ Secondary ⃣ Tertiary

1. **City of Hospital**
2. Riyadh
3. Jeddah
4. **Years of experience**

**⃣** 0-4 years ⃣ 5-9 years ⃣ 10- 14 years ⃣ 15-19 years ⃣ ≥ 20 years

**Section 2: Knowledge**

1. **To what extent do you consider yourself knowledgeable about off-label drug use?**

**⃣**  Excellent **⃣** Average ⃣ Poor

1. Off-label drug use is

| **Using a licensed drug in a manner not mentioned in the drug labeling information** | **Using a drug that is unlicensed** | **Using a drug other than what is mentioned in drug information textbooks** |
| --- | --- | --- |

1. Off-label drug use is common

| **Yes** | **No** | **Don’t know** |
| --- | --- | --- |

1. Can an off-label drug use for a given drug become a widely accepted practice or even a standard of care

| **Yes** | **No** | **Don’t know** |
| --- | --- | --- |

1. In which age group did you think off-label drug use is most common

| **Children** | **Geriatrics** | **Pregnant and lactating women** |
| --- | --- | --- |

1. Which of the following reference indicates that the use of a drug is considered off-label

| **Micromedex** | **Clinical trials. Gov** | **National Guidelines** |
| --- | --- | --- |

1. The major concern regarding the trending increase of the off label drug use is

| **Patient harm** | **Treatment failure** | **Both** |
| --- | --- | --- |

1. In your opinion, how safe are prescribing off-labeled drugs

| **Can or not be Safe** | **Unsafe** | **Safe** |
| --- | --- | --- |

1. The risk/benefit information available on the off-label use of drugs is appropriate

| **No** | **Yes** | **I don’t kwon** |
| --- | --- | --- |

1. Pharmacy and Therapeutic (P & T) Committee…

| **Controlling the off-label use of drugs** | **Its approval is required in every single case** | **Has no role in off-label drug prescribing** |
| --- | --- | --- |

1. Can a pharmaceutical company make a promotion for an unapproved indication

| **No** | **Yes** | **I don’t know** |
| --- | --- | --- |

1. Can a pharmaceutical company speak about off-label use during CME activities

| **Yes** | **No** | **Don’t know** |
| --- | --- | --- |

1. Insurance companies easily pay for off-label use

| **No** | **Yes** | **I don’t know** |
| --- | --- | --- |

1. If a drug is effective, the major obstacle to obtaining government approval to convert its off-label uses to on-label uses is …

| **The cost of obtaining approval and the time needed to enter the market.** | **Insufficient access to scientific references.** | **Ambiguities in regulations of the drug regulatory agencies for obtaining approval** |
| --- | --- | --- |

**Section 3: Clinical Practice**

1. To what extent do you rate yourself in understanding & take required actions in dealing with off-label drug use prescription

| **Excellent** | **Average** | **Poor** |
| --- | --- | --- |

1. Pharmacists have the authority to refuse to fill a prescription if they believe it is incorrect, even if a physician insists on it.

| **Yes** | **No** | **Don’t know** |
| --- | --- | --- |

1. If you received an off-label prescription, the appropriate action will be taken is

| **Contacting the physician to support the use with appropriate evidence & then evaluated by a qualified pharmacist** | **Informed consent from the patient must be obtained after approval of P & T committee before dispensing** | **Refusing to give the drug is the correct action** |
| --- | --- | --- |

1. Gabapentin has been labeled used in post-herpetic neuralgia while it is used as an off-label in treatment of the diabetic neuropathy

| **Yes** | **No** | **Don’t know** |
| --- | --- | --- |

1. There is a law or regulation regarding off-label drugs in the Kingdom of Saudi Arabia released by the SFDA

| **Yes** | **No** | **Don’t know** |
| --- | --- | --- |
